# Supplementary material for: Deletion of the col-26 Transcription Factor Gene and a Point Mutation in the exo-1 F-Box Protein Gene Confer Sorbose Resistance in Neurospora crassa
Source: J Fungi (Basel). 2022 Nov 6;8(11):1169. doi: 10.3390/jof8111169 (PMC9697653; doi:10.3390/jof8111169)
Supplement: Supplementary file 1 [file jof-08-01169-s001.zip › jof-1989961-supplementary.pdf]

Supplementary Materials

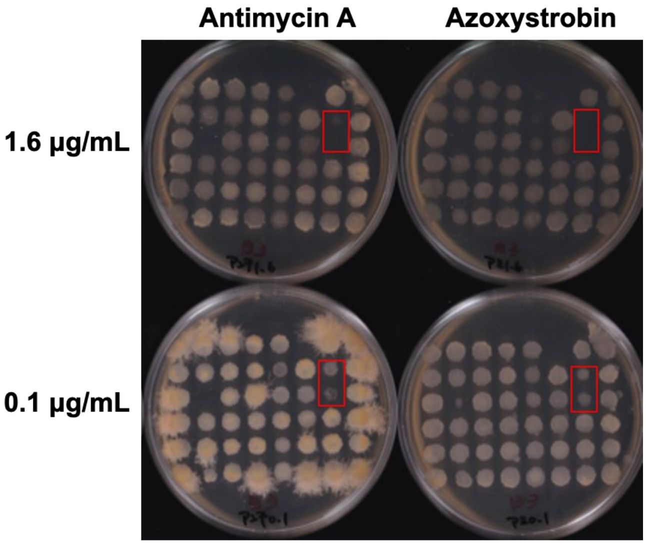

Figure S1 Screening of transcription factor knockout library for sensitivity to the mitochondria complex III inhibitors, azoxystrobin and antimycin A. Conidia of deletion mutants of transcription factors were spotted on SOR medium containing antimycin A and azoxystrobin at 1.6 mg/L and 0.1 mg/L and cultured for 3 days. Red rexes indicate *Δcol-26a* (FGSC#11030) and *Δcol-26A* (FGSC#11031).

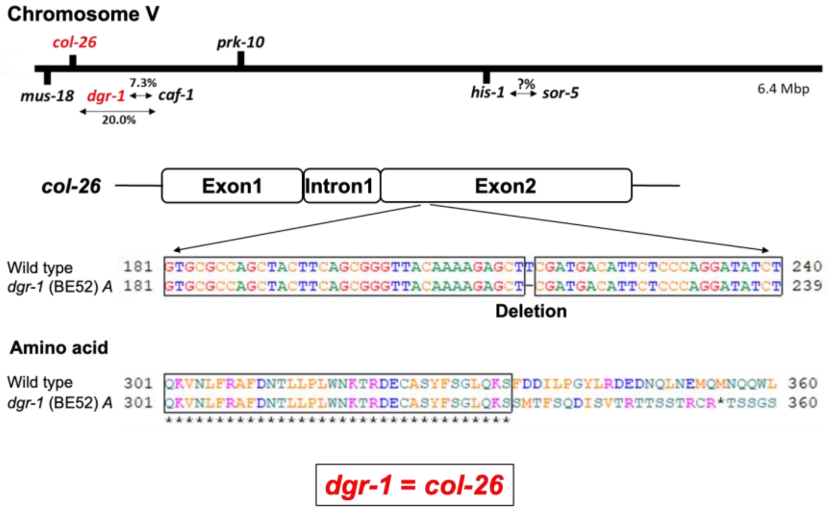

A

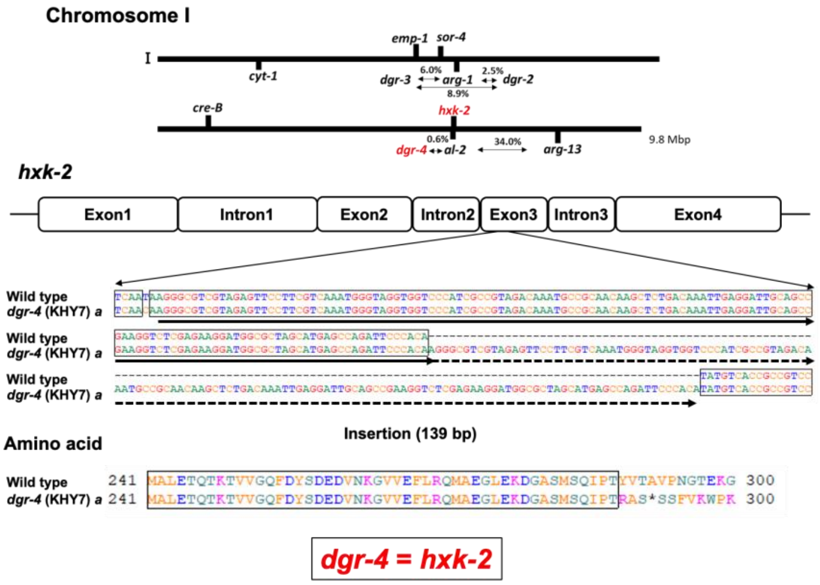

B

Figure S2. S2A Physical and genetical mapping of the *col-26* and *dgr-1* genes, and the mutation of dgr-1(BE52)A strain. The *col-26* gene of the *dgr-1*(BE52)*A* strain was amplified by PCR and sequenced. Deletion of single nucleotide in exon 2 results in a frame shift at amino-acid position 335 of COL-26. This indicates that *col-26* and *dgr-1* are allelic genes. **S2B Physical and genetical mapping of the *hxk-2* and *dgr-4* genes, and the mutation of the *dgr-4* (KHY7) *a* strain.** The *hxk-2* gene of the *dgr-4*(KHY7) *a* strain was amplified by PCR and sequenced. The insertion of 139 bp in exon 3 results in a frame shift at amino-acid position 289 of HXK-2. The solid line indicates the identical sequence to the inserted fragment (dashed line).

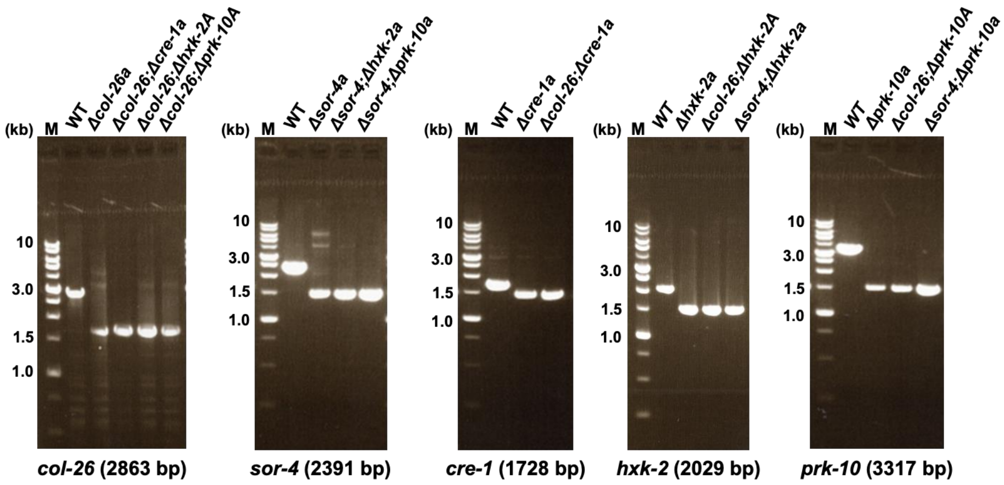

Figure S3 PCR confirmation of the deletion of *cre-1* and *col-26* genes in *Δcol-26;Δcre-1* double mutants. The regions of each gene were amplified by using primer set: *col-26*_F1-*col-26*_R4, *sor-4*_5RC-*sor-4*_3FC, *cre-1*_5RC-*cre-1*_3FC, *hxk-2*_5RC-*hxk-2*_3FC and *prk-10*_5RC-*prk-10*_3FC. The sizes of *col-26*, *sor-4*, *cre-1*, *hxk-2* and *prk-10* in wild-type are 2.9, 2.4, 1.7, 2.0 and 3.3 kb, respectively. The Hyg^r gene which was replaced into each targets was amplified each primers. The deletion mutants were confirmed by the size of Hyg^r gene (1.5 kb).

(A)

```
                                     S11L
                                      *
NcEXO1: ----------------MDSLHITEFGSERYLDKINR
FoFrp1: -----MAHHQARTMAMDPPHITEFASERYFEKLSQ
MgEXO1: -------MATPRHISADHLHITEFASRTYFDKLNQ
BcEXO1: ----------------MEPLHLTEFASERYFDKLRQ
TrEXO1: MAEPHHQQHPPRRVAMDPPHLTEFASERYFEKLNQ
```

(B)

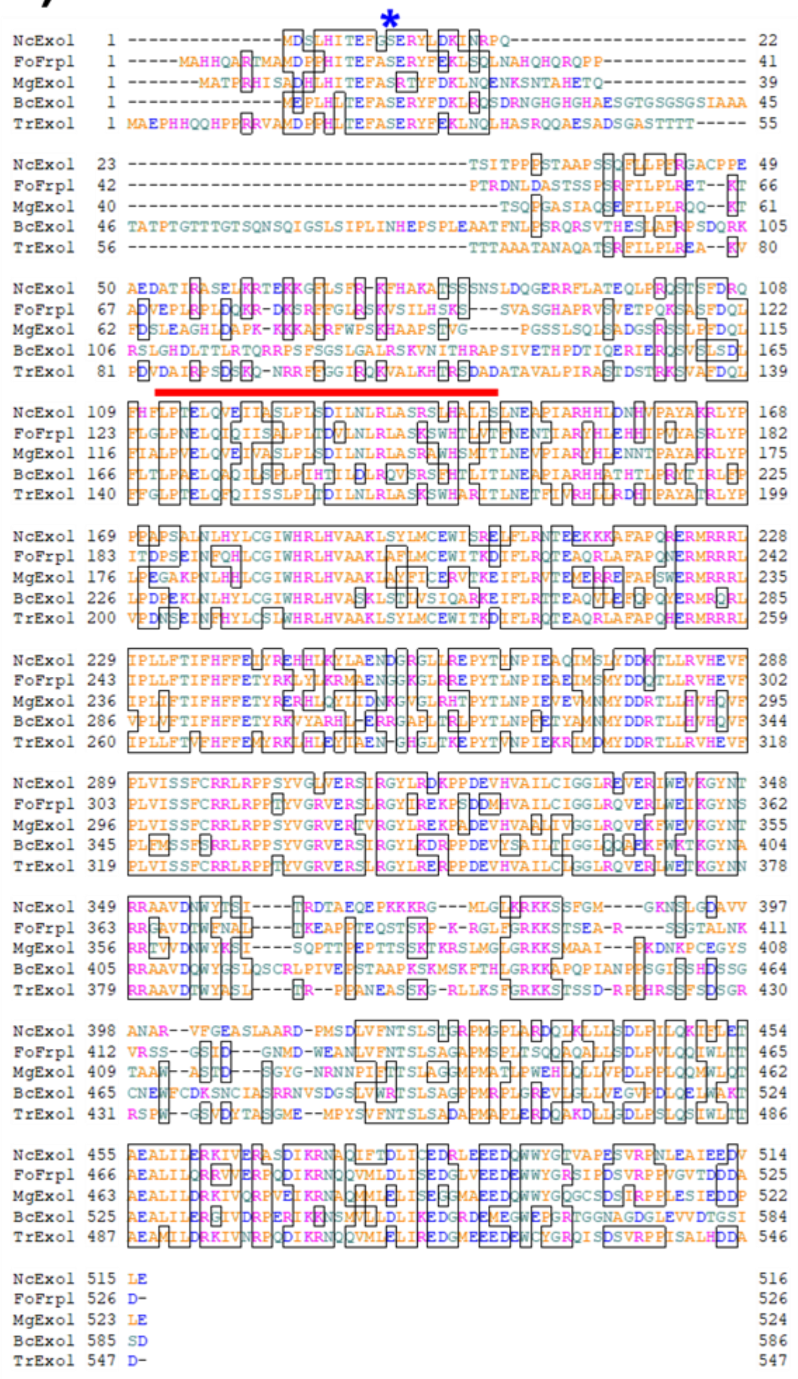

Figure S4. S4A Amino-acid homology of N-terminal region of F-box protein EXO-1 protein in fungi. Asterisk indicates the position of S11L mutation in *dgr-2*(L1)*a* strain. Red letters indicate conserved amino acids. **Figure S4. S4B Alignment of EXO-1 amino-acid sequences in fungi. The *dgr-2*(L1)*a* strain has S11L mutation in EXO-1**. Asterisk indicates the position of S11L mutation. F-box domain (112 to 144) is indicated

by red line. *Neurospora crassa* NcEXO-1 (ACCESSION; EAA29388.2), *Fusarium oxysporum* FoFrp1 (AAT85969) *Magnaporthe grisea* MgEXO-1(EHA50910), *Botrytis cinerea* BcEXO1 (XP_024551558), *Trichoderma reesei* TrEXZO1 (EGR50794)

Table S1. List of sequencing primers for *col-26* and *hxk-2* genes

Primer name	Sequence (5'→3')
col-26_seq_F1	TGTCTGTCTACCTACCTACC
col-26_seq_F2	TGAATGGCATCAACAGCCCG
col-26_seq_F3	AATGGATCCTGTGTAGAGCC
col-26_seq_F4	ACAAGTTCAGTCTTCCTCGC
col-26_seq_F5	ACAGCAAATACGGACCGTGG
col-26_seq_R1	AGCACACAGTATGTGTCGAC
col-26_seq_R2	TCGTCACGGAGATATCCTGG
col-26_seq_R3	TGGAGACATGATGGAAGGGG
col-26_seq_R4	TGTAGCCATGTTTCCCGAGC
hxk-2_seq_F1	AAGACGCAAAATAACCCCGC
hxk-2_seq_F2	TTACCGGCATTTTGCCTTGG
hxk-2_seq_F3	ACAAAGACCGTTGTCGGTCAG
hxk-2_seq_F4	AACATCAAGAAGCCCCTAGC
hxk-2_seq_F5	ATTGCTGGTGCTATCGGTCG
hxk-2_seq_R1	CAGGAAAGCTGCACTTGTCC
hxk-2_seq_R2	TTTGTCAGAGCTTGTTGCGG
hxk-2_seq_R3	ATGGTGTGTTAGGAAGGACC
hxk-2_seq_R4	ACAATGCACCTCTTTCTGCG

Table S2. List of primers used for PCR detection of gene deletions

Primer name	Sequence (5'→3')
col-26_seq_F1	TGTCTGTCTACCTACCTACC
col-26_seq_F4	ACAAGTTCAGTCTTCCTCGC
sor-4_5RC	GCATACAGCCTACAGTCAAG
sor-4_3FC	CCAGAAAGCTCGAGGGAAAT
cre-1_5RC	GGTCCGGTCTACAATCTC
cre-1_3FC	GCTCCTAGTCAAGAACCAG
hxk-2_5RC	CCACTTTGACTCCTCTCACA
hxk-2_3FC	CGTTCATCCTTACACAGCAC
prk-10_5RC	CCTCCTATGGAACCTAACAG
prk-10_3FC	GGTGCATGATGAGACATAGC

Table S3. List of RT-qPCR primers and universal probes

Primer name	Target gene	Sequence (5'→3')	Probe#
gla-1_L	NCU01517	CTACGACGCCGTCTACGTCT	10
gla-1_R	NCU01517	AGGGAACGAGGTCCTTGAA	10
gh13-2_L	NCU09805	CGTGAGTTGCGATAGCTTCA	16
gh13-2_R	NCU09805	CCCCAAAAAGTGTGATGGTTA	16
gh31-3_L	NCU04674	CCTGCCGTAAGGACACCTC	11
gh31-3_R	NCU04674	CCTGCCGTAAGGACACCTC	11
inv_L	NCU04265	GGGGTCCCATGTACTGGTC	47
inv_R	NCU04265	GTGTCCTGCGTGTTGTTGAC	47
glt-1_L	NCU01633	CAGCAACTCCGCCTCTTG	12
glt-1_R	NCU01633	ATATTCCGCCACCGAGAAC	12
hgt-1_L	NCU10021	AGGAGTACGAGCGTCAGCTT	22
hgt-1_R	NCU10021	AGAACCCTTGAAGCAGTTGG	22
act_L	NCU04173	GCTGAGCGTGGTTACACCTT	77
act_R	NCU04173	TCCTTGATGTCACGAACGAT	77
